# The role of the gut microbiota in the dietary niche expansion of fishing bats

**DOI:** 10.1186/s42523-021-00137-w

**Published:** 2021-10-28

**Authors:** Ostaizka Aizpurua, Lasse Nyholm, Evie Morris, Gloriana Chaverri, L. Gerardo Herrera Montalvo, José Juan Flores-Martinez, Aiqing Lin, Orly Razgour, M. Thomas P. Gilbert, Antton Alberdi

**Affiliations:** 1 Center for Evolutionary Hologenomics, GLOBE Institute, University of Copenhagen, 1353 Copenhagen, Denmark; 2 University of Exeter, Streatham Campus, Biosciences, Exeter, EX4 4PS UK; 3 Sede del Sur, Universidad de Costa Rica, #4000 Alamedas, Golfito, 60701 Costa Rica; 4 Smithsonian Tropical Research Institute, Apartado 0843-03092, Balboa, Ancón República de Panamá; 5 Estación de Biología Chamela, Instituto de Biología, Universidad Nacional Autónoma de México, Apartado Postal 21, San Patricio, 48980 Jalisco, Mexico; 6 Laboratorio de Sistemas de Información Geográfica, Departamento de Zoología, Instituto de Biología, Universidad Nacional Autónoma de México, Circuito Exterior s/n, Ciudad Universitaria, 04510 Mexico City, Mexico; 7 Jilin Provincial Key Laboratory of Animal Resource Conservation and Utilization, Northeast Normal University, Changchun, 130117 China; 8 University Museum, Norwegian University of Science and Technology, 7491 Trondheim, Norway

**Keywords:** Chiroptera, Diet, Microbiome, Microorganism, Niche shift, Piscivorous, Trophic niche

## Abstract

**Background:**

Due to its central role in animal nutrition, the gut microbiota is likely a relevant factor shaping dietary niche shifts. We analysed both the impact and contribution of the gut microbiota to the dietary niche expansion of the only four bat species that have incorporated fish into their primarily arthropodophage diet.

**Results:**

We first compared the taxonomic and functional features of the gut microbiota of the four piscivorous bats to that of 11 strictly arthropodophagous species using 16S rRNA targeted amplicon sequencing. Second, we increased the resolution of our analyses for one of the piscivorous bat species, namely *Myotis capaccinii,* and analysed multiple populations combining targeted approaches with shotgun sequencing. To better understand the origin of gut microorganisms, we also analysed the gut microbiota of their fish prey (*Gambusia holbrooki*). Our analyses showed that piscivorous bats carry a characteristic gut microbiota that differs from that of their strict arthropodophagous counterparts, in which the most relevant bacteria have been directly acquired from their fish prey. This characteristic microbiota exhibits enrichment of genes involved in vitamin biosynthesis, as well as complex carbohydrate and lipid metabolism, likely providing their hosts with an enhanced capacity to metabolise the glycosphingolipids and long-chain fatty acids that are particularly abundant in fish.

**Conclusions:**

Our results depict the gut microbiota as a relevant element in facilitating the dietary transition from arthropodophagy to piscivory.

**Supplementary Information:**

The online version contains supplementary material available at 10.1186/s42523-021-00137-w.

## Background

Given their fundamental roles in acquiring the energy needed for animals to develop, survive and reproduce, traits associated with diet are expected to be under strong selection pressure [1]. Thus, diversification of dietary niches is one of the prevailing processes in animal evolution [2]. However, when a novel dietary resource differs considerably from the original, the resulting trophic change might need to be accompanied with a physiological adaptation, so that animals can make the most of the nutritional value of the novel food [3]. The gut microbiota has been identified as a key element for such processes [4, 5], first, because microorganisms can complement the digestive capabilities of the host by extracting and metabolising dietary ingredients that the hosts’ enzymatic toolbox are unable to process; and second, because microorganisms provide essential compounds like vitamins and short-chain fatty acids to their hosts [6, 7]. Many microorganisms that reside in the intestinal tract of animals are acquired directly through the diet [8], and some contribute to the metabolism of dietary ingredients, thus providing the host with the capacity to better exploit the nutritional potential of the food [9].

**Fig. 1:**
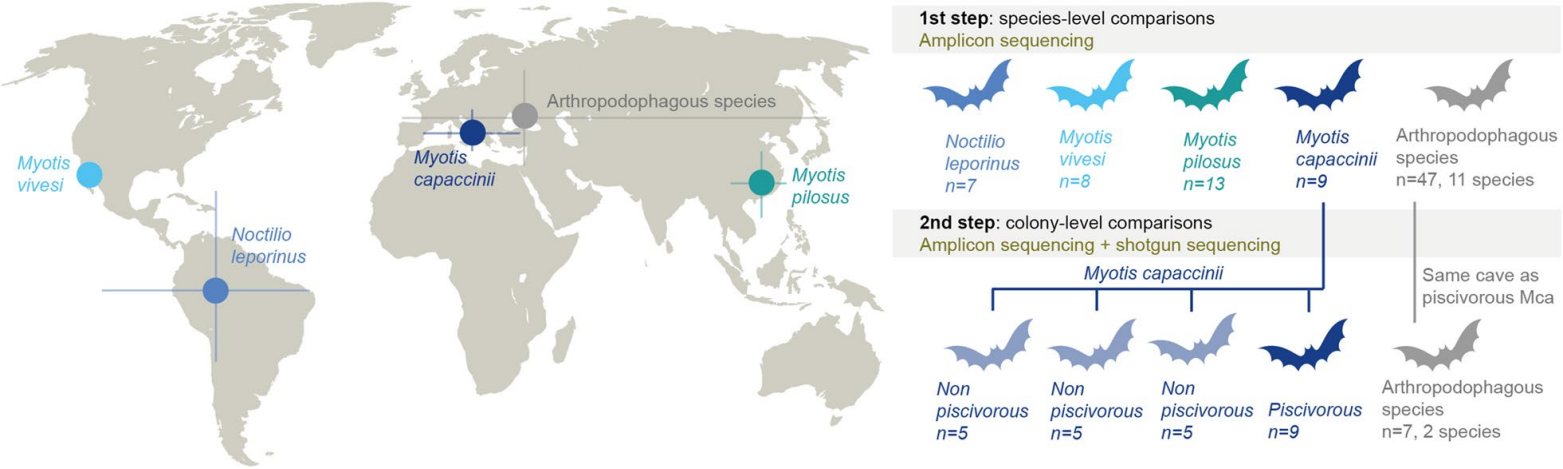
Map of the approximate distributional range (latitude and longitude limits) of bats analysed in this study (left), and species and number of individuals (n) used in each step of the analysis (right). In the first step, 37 bats from four well-known fish-consuming colonies of the four piscivorous species were compared to 47 bats belonging to 11 strict arthropodophagous species. In the second step, we increased the resolution of the analyses in the piscivorous bat *Myotis capaccinii*, by adding more individuals (n = 15) from three allegedly non-piscivorous colonies to the analysis. For these analyses we also included arthropodophagous bats that roost in the same cave as the piscivorous *M. capaccinii, namely Miniopterus schreibersii* and *Myotis myotis* as controls

Bats are an excellent system with which to study the role of microorganisms in such dietary shifts, due to recurrent invasion into non-arthropod feeding niches [10–12], and their digestive capabilities. Despite having reduced the length of their intestinal tract as a weight-saving adaptation to flight, the digestion and absorption capacities of bats are comparable to those of similar-sized non-flying mammals [13]. While some bat species have specialised in consuming a single food resource (e.g. blood-feeding bats, [14]), others have incorporated new feeding resources into their ancestral arthropod-based diet [10], thus expanding their dietary niche. One example of the latter is the case of the four well-known piscivorous (also known as fishing) bat species [15], which have independently developed fishing behaviour in four geographical areas across the planet (Fig. 1). However, the four species exhibit different patterns of fish consumption, ranging from widespread (occurring in most of the individuals) and common (up to 90%) in *Noctilio leporinus*, to restriction to only certain colonies and seasons in the three species of *Myotis* piscivorous bats; namely, *M. pilosus*, *M. vivesi* and *M. capaccinii* [15]. Despite this fascinating twist to their life history, the spatio-temporal patterns, causes and consequences of fishing behaviour largely remain unexplored.

Given there are considerable nutritional differences between arthropods and fish [16], we predicted that piscivorous bats host a gut microbiota that is distinct from that of their strictly arthropodophagous (i.e. organisms that prey on arthropods, [17]) counterparts. Our rationale is based on the hypothesis that this microbiota could help confer the digestive capability required to acquire nutrients from fish flesh. We also explore whether the new repertoire of microbial functions has been acquired from microorganisms associated with the new food resource. Finally, as piscivory is often limited to certain colonies, and perhaps certain individuals, rather than established among all individuals within a species [15], we also analysed whether microbiota traits associated with piscivory are restricted to actively fishing individuals, or established across populations of piscivorous bats. Overall, we aim to understand not only how a dietary shift affects gut microbial communities, but also how microbiota changes can facilitate such a trophic niche expansion.

## Results and discussion

We first used targeted amplicon sequencing of the bacterial 16S rRNA gene to generate and analyse the taxonomic and functional gut microbiota profiles of 47 individuals belonging to 11 strict arthropophagous bat species and 37 individuals belonging to well-known piscivorous colonies of the four facultative piscivorous species (e.g. consuming both arthropods and fish, hereafter referred to simply as piscivorous for the sake of clarity) (Fig. 1, see Additional file 1: Table S1.1). In a second step, we increased the resolution of our analyses in the piscivorous bat *Myotis capaccinii,* by adding more individuals from three allegedly non-piscivorous colonies to the analysis, as well as incorporating shotgun sequencing data for direct fish DNA quantification and inference of functional microbiota features. To gain further insights into the origin of gut microorganisms, we also analysed the gut microbiota of *M. capaccinii*’s fish prey (*Gambusia holbrooki*). We then implemented an integrative approach that included Hill numbers-based diversity analyses [18], multivariate statistics, ensemble machine learning modelling [19] and enrichment analyses, in order to (1) identify the gut microbiota patterns behind this trophic shift, (2) unveil the factors shaping gut microbial communities, and (3) understand the implications of hosting different microbiotas for bats.

### Piscivorous bats host unique gut microbiotas

**Fig. 2:**
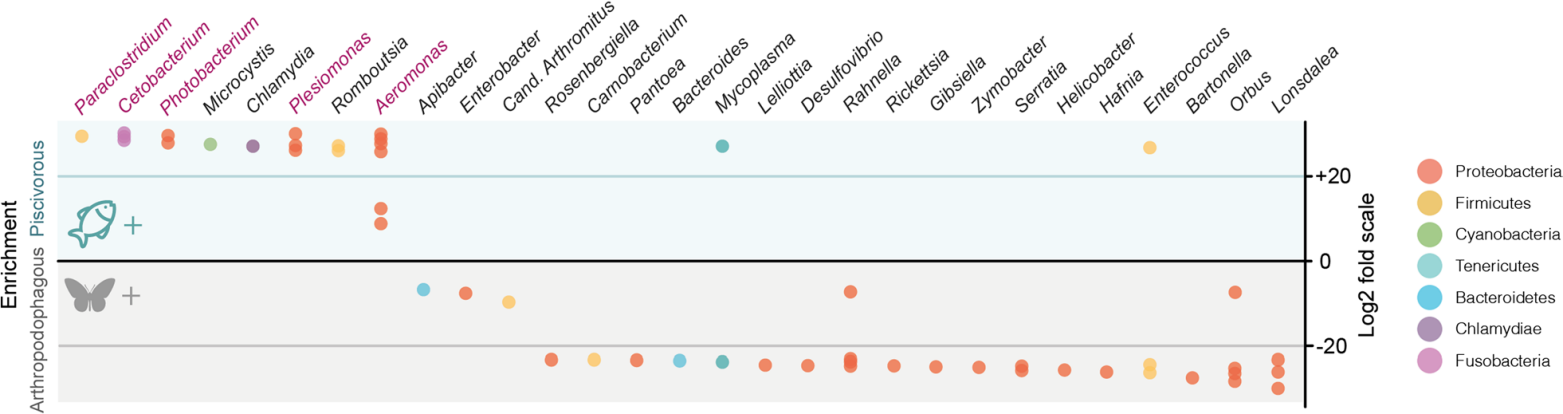
Differentially abundant amplicon sequence variants (ASVs) between piscivorous and arthropodophagous bats displayed at the bacterial genus level. Only ASVs with a significance value of *p* < 0.01 are shown. Positive values indicate genera that were enriched in piscivorous bats, while negative values show those enriched in arthropodophagous bats. Colours of the circles indicate phyla. Each circle represents a single ASV, thus multiple circles within a genus indicate multiple ASV that were enriched. The five highlighted bacterial genera are the ones that contributed the most to the ensemble predictive models of piscivorous bats

Taxonomic characterisation of the gut microbiota associated with the 15 bat species studied using amplicon sequencing exhibited an overall microbial community that was comprised of 27 phyla, which was principally dominated by Proteobacteria (56.36 ± 33.41%; mean ± standard deviation) and Firmicutes (22.16 ± 28.78%) (see Additional file 1: Fig. S1.1). These overall patterns are in accordance with previous observations [20–23]. We observed large interindividual (overall amplicon sequence variant (ASV) turnover rate across individuals within species of 87.98 ± 0.08%) and interspecific variability (PERMANOVA_U12_: R^2^ = 0.195, *p* value = 0.001; PERMANOVA_Ū12_: R^2^ = 0.198, *p* value = 0.001, see Additional file 1: Table S1.4) in their gut microbiota, indicating the community likely responds to the wide breadth of ecological and evolutionary features embedded within the animals studied. However, this variability did not mask diet-related patterns. In particular, despite exhibiting similar microbial diversity values (Wilcoxon: *p* value > 0.05 at different q values, see Additional file 1: Table S1.2 and Fig. S1.2), both the analysis of variance (PERMANOVA_U12_ R^2^ = 0.057, *p* value = 0.001; PERMANOVA_Ū12_: R^2^ = 0.072, *p* value = 0.001, see Additional file 1: Table S1.4 for PERMANOVA analysis on other neutral and phylogenetic Hill number of order of diversity 0) and the ensemble machine learning classification (accuracy = 1) showed that piscivorous bats have compositionally characteristic gut microbial communities that differ from that of arthropodophagous bats. The distinctiveness of piscivorous bats is mainly attributed to five bacteria genera, namely *Aeromonas, Plesiomonas, Photobacterium, Cetobacterium* and *Paraclostridium*, as these were significantly enriched in piscivorous bats (Fig. 2), and were also the bacteria that contributed the most to the ensemble predictive models of piscivorous bats (see Additional file 1: Table S1.3).

### The gut microbiota of piscivorous bats shows limited signature of convergence

**Fig. 3:**
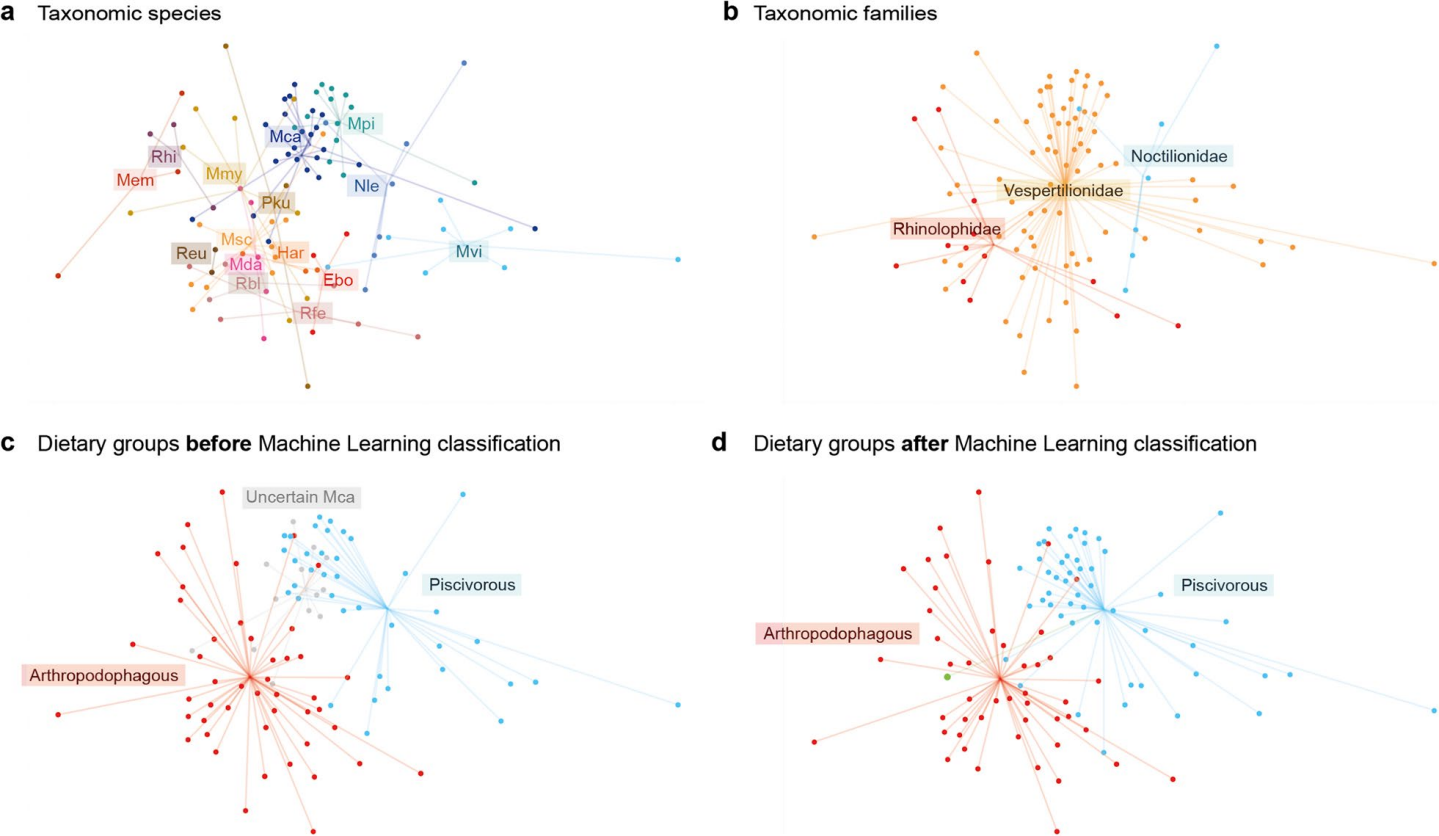
Ordination of the gut microbial communities of the analysed bats. **a** Samples coloured by host species. **b** Samples coloured by taxonomic families. **c** Samples coloured according to the dietary groups set before the machine learning classification. Grey dots represent *Myotis capaccinii* individuals from colonies in which no piscivory has been reported. These samples were excluded from the machine learning model training process. **d** Samples coloured according to the dietary groups predicted by the machine learning classification. The green dot is the only *M. capaccinii* that was classified as an arthropodophage. The bat species abbreviations are Ebo = *Eptesicus bottae*, Har = *Hypsugo ariel*, Msc = *Miniopterus schreibersii*, Mca = *Myotis capaccinii*, Mda = *M. daubentonii*, Mem = *M. emarginatus*, Mmy = *M. myotis*, Mpi = *M. pilosus*, Mvi = *M. vivesi*, Nle = *Noctilio leporinus*, Pku = *Pipistrellus kuhlii*, Rbl = *Rhinolophus blasii*, Reu = *R. euryale*, Rhi = *R. hipposideros*, Rfe = *R. ferrumequinum*

We did not detect a core microbiota (i.e. bacteria represented in > 90% of individuals) at ASV- or genus-level, among piscivorous or among arthropodophagous bats. Accordingly, the relative representation of bacterial taxa characteristic of piscivorous bats differed markedly across species, ranging from marginal presence in some, to being the dominant taxon in others. Consequently, while the resulting gut microbiotas of piscivorous bats were different from those of arthropodophagous species, they did not converge into one characteristic type of microbial community associated with piscivory (PERMANOVA_U12_: R^2^ = 0.312, *p* value = 0.001, Fig. 3, see Additional file 1: Table S1.4). Similar patterns have been described as convergence in other groups of phylogenetically distant species with similar diets, such as ant-eating mammals [24]. However, we argue that convergence entails not only exhibiting distinctive microbial communities, but also an increase in their similarity, something that is not observed in any of the cases. Extending our work by generating deep shotgun metagenomic data from the different fishing bat species would enable ascertaining whether the observed microbiota variations entail functional convergence [25, 26].

**Fig. 4:**
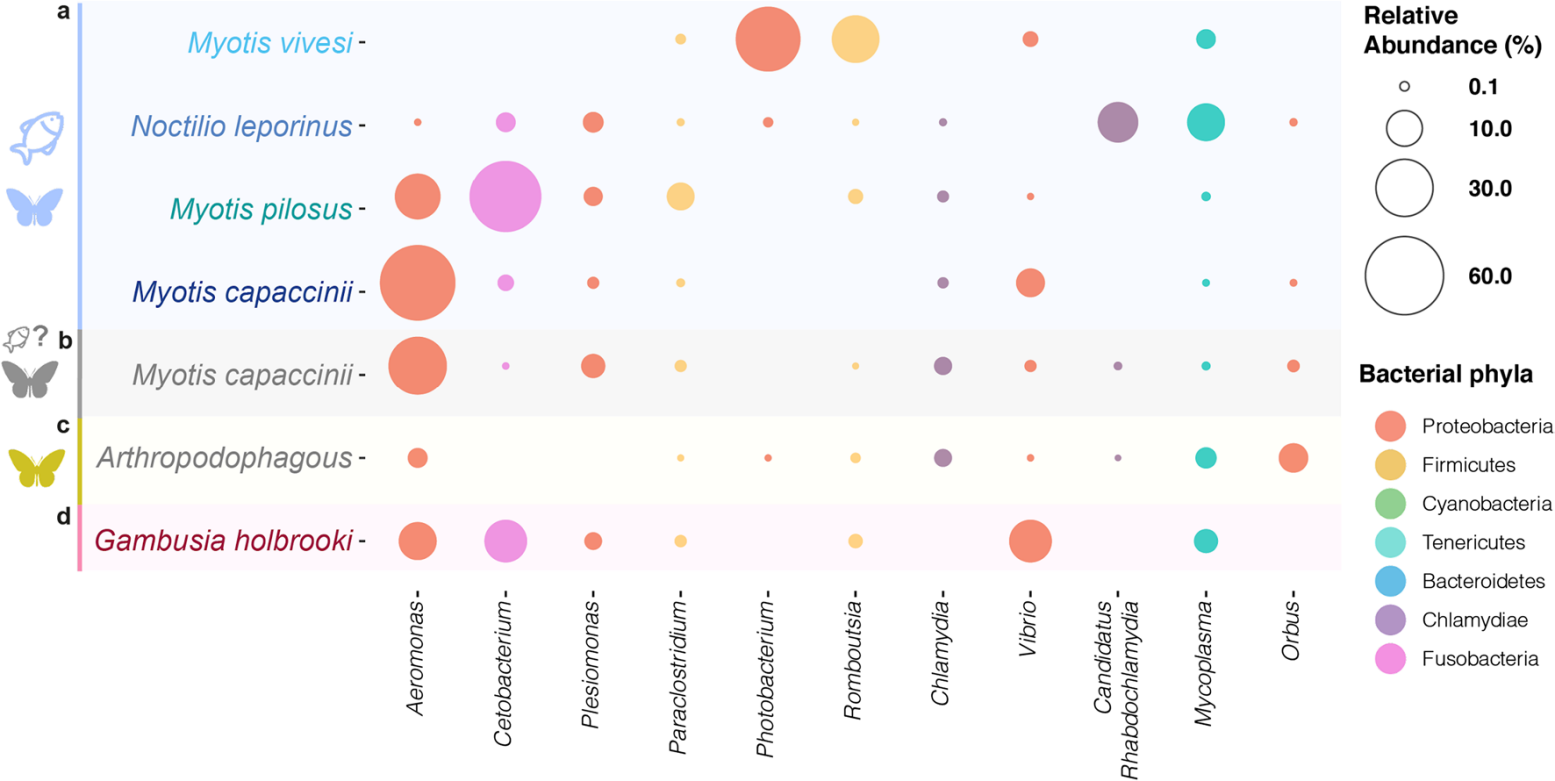
Relative abundance of the most relevant bacteria enriched in piscivorous or arthropodophagous bats in **a** the four piscivorous bat species, **b** *Myotis capaccinii* colonies in which no piscivory has been recorded so far, **c** the eleven arthropodophagous bats analysed, and **d** the eastern mosquitofish (*G. holbrooki*) consumed by piscivorous *M. capaccinii*

The two piscivorous species with the highest microbial community resemblance are *M. capaccinii* and *M. pilosus* (Fig. 3a, see Additional file 1: Fig. S1.3), due to the high representation of *Aeromonas* and *Cetobacterium* in both species (Fig. 4). *M. capaccinii* and *M. pilosus* are the two piscivorous bats with both the highest phylogenetic [27] and ecological resemblance, as they exclusively forage in freshwater habitats [28, 29]. However, the reason for the dominant bacteria to be different in each bat —*Aeromonas* (Proteobacteria) in *M. capaccinii* and *Cetobacterium* (Fusobacteria) in *M. pilosus*— might be that the bats inhabit different habitats (*M. capaccinii* is found in Mediterranean and *M. pilosus* in temperate-subtropical habitats), and consume different species of fish [29, 30].

The other two piscivorous bat species exhibited rather different microbial communities, probably shaped by different extrinsic and intrinsic forces. The microbiota of *M. vivesi* stands out for an overall dominance of Firmicutes (49.07 ± 42.15%, see Additional file 1: Fig. S1.1), a high representation of *Photobacterium* (Proteobacteria), and an absence of *Aeromonas* and *Cetobacterium* (Fig. 4). We believe this relates to the fact that *M. vivesi* is the sole species to specialise in foraging in the ocean [31]. The high salinity of the water and type of prey might modify the physicochemical conditions of the intestinal environment, up to the point of shaping a completely different microbiota [32, 33]. This is supported by observations of higher prevalence of Firmicutes compared to Proteobacteria in salinity gradients [34]. Increased incidence of Firmicutes has also been related to the consumption of the Engraulidae fish [35] that *M. vivesi* consumes [36], due to their high content of polyunsaturated fatty acids that are known to promote intestinal enrichment of Firmicutes [37]. Furthermore, the pool of microbial species is different in salt and fresh water [38], for instance *Photobacterium* are ubiquitous in the oceans [39] and often found in symbiotic relationships with fish within the dietary spectrum of *M. vivesi* [40, 41].

The gut microbiota of *N. leporinus* is also different from that of other piscivorous species, probably due to a combination of the frequency of piscivory, the fish species consumed, the foraging environment, and its evolutionary distinctiveness. The incidence of fish in the diet of *N. leporinus* is considerably larger than in the three *Myotis* species [42]. It can forage over water with different levels of salinity [43, 44], although in the area in which we sampled the bats predominantly forage in brackish water. Accordingly, we detected both the freshwater-related bacteria *Cetobacterium* as well as the seawater-related *Photobacterium* (Fig. 4)*.* In addition, the 50 Myr of independent evolution of Noctilionids and Vespertilionids (to which the other three piscivorous *Myotis* species belong) [45] could have also introduced physiological differences that impede stronger convergent processes.

### Characteristic microbiotas of piscivorous bats are not limited to actively fishing individuals

We assessed whether the piscivory-type gut microbiota is restricted to animals actively consuming fish, or instead extended across populations within a piscivorous species regardless of whether they commonly consume fish or not. To do so, we increased the scope of our analyses in one of the piscivorous bat species, *M. capaccinii*, to compare the gut microbiota of individuals from a well-known piscivorous colony [29] to those sampled at three other colonies in which no evidence of piscivory was detected after screening of bulk guano samples for fish remains, and mining shotgun metagenomic data for fish DNA traces (Additional file 1: Table S1.6).

We found that all piscivory-related bacteria enriched in *M. capaccinii* from the piscivorous colony were also enriched in all individuals from other (no known piscivory) colonies. The permutational multivariate analysis of variance also indicated that there was no difference between the gut microbiota composition of the piscivorous and allegedly non-piscivorous *M. capaccinii* (pairwise PERMANOVA_U12_, R^2^ = 0.065, *p* value_FDR_ = 0.090, see Additional file 1: Fig. S1.4 and Table S1.5). Similarly, the machine learning modelling classified the gut microbiota of all *M. capaccinii* individuals, except one, as communities characteristic of piscivorous bats (Fig. 3d). The fact that almost all *M. capaccinii* exhibit a piscivorous-like microbiota could be indicative of a more widespread consumption of fish than previously thought. However, the screening of faecal material from multiple *M. capaccinii* colonies, through visual inspection of the faecal pellets produced by the analysed individuals and the shotgun-sequencing based DNA analysis (Additional file 1: Table S1.6), showed no traces of piscivory in any of the non-piscivory colonies. An alternative hypothesis could be that this characteristic gut microbiota is the result of an ancestral establishment of piscivory-related bacteria in the gut of this bat species as a remnant of a more widespread fishing behavior in the past.

### The characteristic bacteria of piscivorous bats were likely ancestrally acquired from their fish prey

To gain further insight into the relationship between piscivorous bats and their characteristic gut bacteria, we explored the means of acquisition of piscivory-associated bacteria. The bacterial taxa enriched in piscivorous bats are common bacterial colonisers in the intestinal tract of freshwater and/or marine fish [46–50]. This led us to hypothesise that these taxa could have been acquired from their fish prey. To explore this, we analysed in detail the relationship between microorganisms found in the gut of *M. capaccinii* and their fish prey. For these analyses we also included as contrast arthropodophagous bats that roost in the same cave as the piscivorous *M. capaccinii*, namely *Miniopterus schreibersii* and *Myotis myotis*. We found that only two of the 28 bacteria genera overrepresented in these two species were also present in the gut environment of Gambusia, namely *Mycoplasma* and *Desulfovibrio*. *Mycoplasma* are common bacteria among arthropodophagous bats [51, 52] and *Desulfovibrio* inhabit the intestinal tract of many insects [53], which both bats and Gambusia prey on [54]. In contrast, we observed that all but one of microbial genera overrepresented in piscivorous *M. capaccinii* (the exception being *Alysiella* (Proteobacteria), see Additional file 1: Fig. S1.5) also belong to the gut microbiota of the fish species that this bat colony consumes, i.e. *Gambusia holbrooki* [29]. This led us to conclude that the origin of the bacteria taxa characteristic of piscivorous bats is consistent with acquisition from their fish prey.

**Fig. 5:**
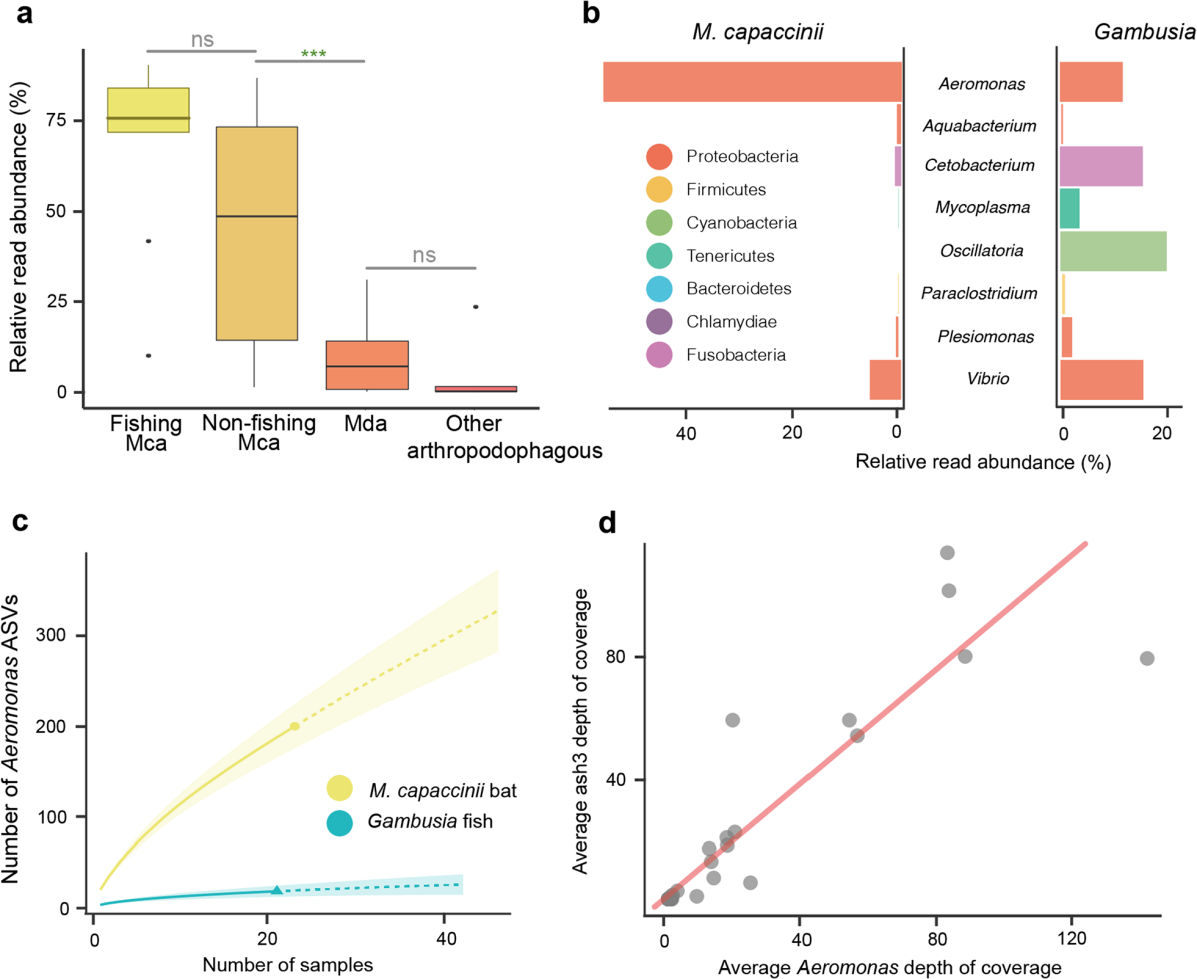
**a** Cumulative relative representation of ASVs detected in fish guts measured in different groups of bats. **b** Relative representation of bacteria in the intestinal tracts of *M. capaccinii* and Gambusia. **c** Number of *Aeromonas* ASVs detected in *M. capaccinii* bats and Gambusia fish. **d** Depth of coverage of *Aeromonas* and ash3 (aerolysin precursor) gene recovered from bat shotgun metagenomes

To explore whether there is a selective mechanism in *M. capaccinii* that determines which of the bacteria acquired from the fish are established in their gut, we compared ASVs identified in *M. capaccinii* with the ASVs found in *G. holbrooki*. We found exact matches of 343 of the ASVs detected in *M. capaccinii* within the intestine of the fish species consumed. The cumulative relative representation of these ASVs was considerably higher in *M. capaccinii* (68.60 ± 26.4% piscivorous *M. capaccinii*, 44.8 ± 30.80% non-piscivorous *M. capaccinii*) than among arthropodophagous bats (5.14 ± 10.30%; K-W_FMcap-Arthrop_: X^2^ = 18.767, df = 3, *p* value = 0.0003, Fig. 5a). These results suggest that many bacteria acquired from fish are not transient taxa that are only detected following recent fish consumption, but there is a filtering mechanism that determines which bacteria are established and actively maintained in the bat intestinal tracts. A similar pattern has also been observed in vultures, where the gut microbiota is conserved between captive-bred and wild individuals despite having different diets [55]. The differences between the relative representation of bacteria in the Gambusia and *M. capaccinii* intestinal tracts also support such a *selective* acquisition of microorganisms. The most abundant —yet most likely transitory— microbial taxon in Gambusia, namely the cyanobacteria *Oscillatoria*, was not detected in the gut of *M. capaccinii*, and the representation of another of the most abundant taxa in Gambusia, namely *Mycoplasma*, was marginal in piscivorous bats. In contrast, the representation of *Aeromonas* was five times larger in piscivorous bats than in Gambusia (Fig. 5b). Furthermore, the bacterial replication rate estimates from shotgun metagenomic data indicated that *Aeromonas* are actively replicating in the bats' gut, with no significant differences between piscivorous and non-piscivorous *M. capaccinii* (Additional file 1: Table S1.6). *Aeromonas* were not only found to be highly abundant and actively replicating in the intestine of *M. capaccinii*, but we also detected an extreme diversity. After studying a similar number of bat and fish individuals with the exact same methodology, the number of *Aeromonas* ASVs detected among piscivorous bats was ten times higher than in Gambusia (Fig. 5c). This points to the presence of multiple *Aeromonas* strains in bat intestines that are currently absent, or are very uncommon, in Gambusia populations around the piscivorous *M. capaccinii* colony. These could have been acquired from chironomids [56], which are the most consumed arthropod taxon by *M. capaccinii* [57], as *Aeromonas* are common gut bacteria of these arthropods [56]. However, this would not explain the differences observed between *M. capaccinii* and *M. daubentonii*, as the latter also heavily consumes chironomids [58] (Fig. 5a). Thus, the observations support the aforementioned hypothesis whereby *Aeromonas* would have been ancestrally acquired from fish and transferred across colonies and generations while accumulating genetic variation.

### *Myotis capaccinii* are not negatively affected by enterotoxic *Aeromonas*

The high incidence and abundance of *Aeromonas* among piscivorous bats is striking, because most *Aeromonas* strains contain virulence genes responsible for encoding enterotoxins that produce gastrointestinal diseases in fish and humans [59]. To gain insights into the potential pathogenicity of *Aeromonas* in the bat’s guts, we screened for the presence of genes encoding for enterotoxins in the metagenomic assembly. We detected the presence of the virulence gene *ash3* encoding for the toxin aerolysin with a three amino acid difference to the curated reference sequence of the gene characterised from *Aeromonas salmonicida* (UniProKBt/Swiss-Prot Q08676). The average depth of coverage for this gene was similar to the average depth for *Aeromonas* (t-test_paired_: t = 0.096, df = 45, *p* value = 0.92; Fig. 5d), which indicates that the most abundant *Aeromonas* strains in the bat microbiota carry the gene encoding for the enterotoxin aerolysin. This toxin has the capacity to bind to eukaryotic cells and aggregate to form pores in the cell membrane leading to osmolysis, and it has also been shown to facilitate the invasion of more *Aeromonas* [60]. The high abundances of *Aeromonas* bacteria in the gut of *M. capaccinii* could a priori indicate such a pathogenic scenario, although the diversity and prevalence of *Aeromonas*, as well as the fact that all captured animals showed no external signatures of sickness (e.g. no diarrhea, good fur appearance, normal behaviour), suggests that either *Aeromonas* do not heavily express the *ash3* gene in the bat gut, bats have mechanisms to protect their intestinal walls against aerolysin [61] or others members of the microbiota are acting to suppress or modulate the expression of the virulence gene of *Aeromonas* [62].

### Do piscivory-related bacteria provide nutritional benefits to bats?

To better understand the role of piscivory-related bacteria in the gut of piscivorous bats, we explored whether the characteristic gut microbiotas exhibited by piscivorous bats might provide nutritional benefits to their hosts. To do this we implemented a dual approach consisting of (1) a Piphillin-based functional prediction [63] using the entire amplicon sequencing dataset comprising all piscivorous and arthropodophagous species, and a (2) direct shotgun sequencing-based functional profiling in a subset of piscivorous (*M. capaccinii*) and arthropodophagous bats.

**Fig. 6:**
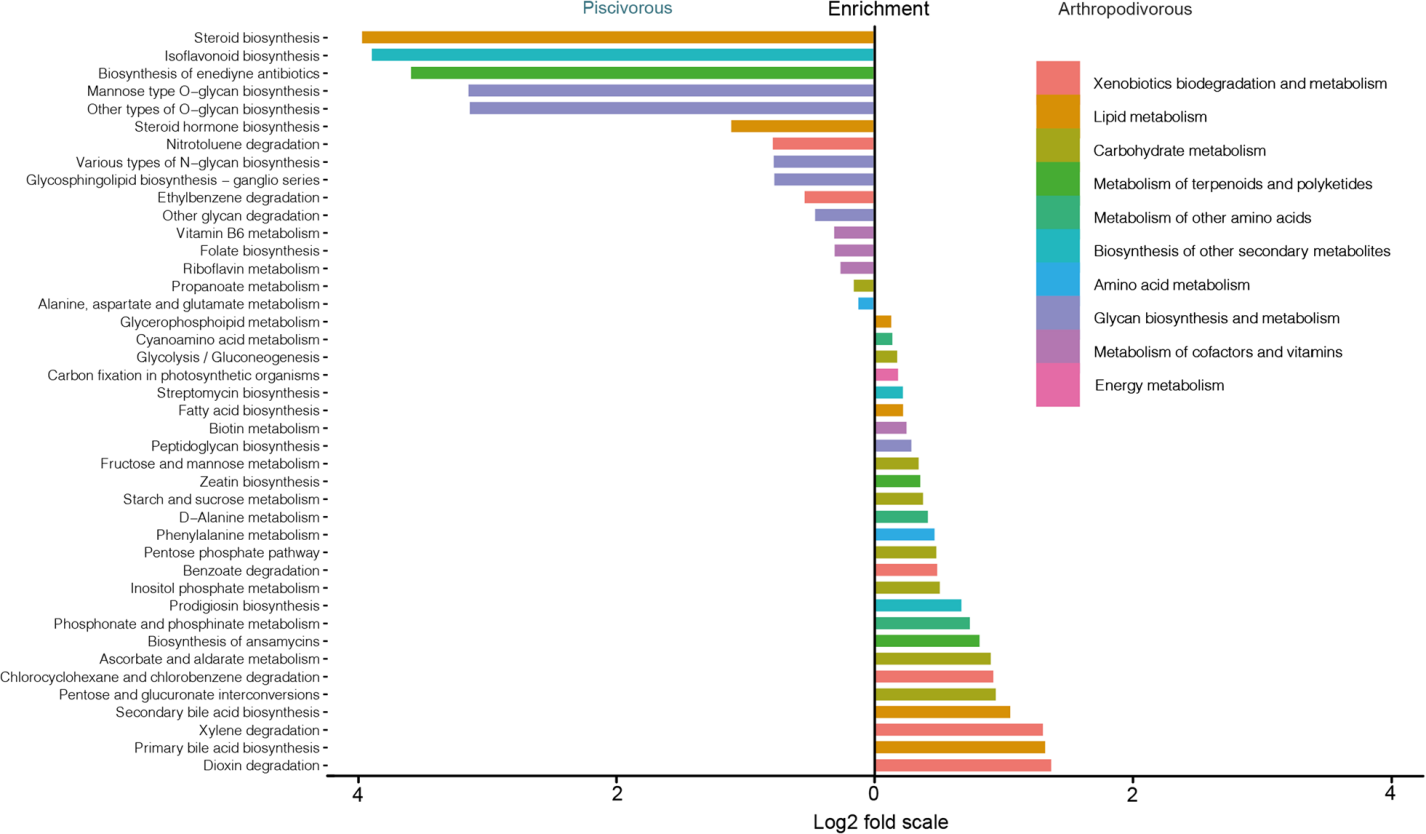
Differential abundance analysis of Piphillin predicted KEGG pathways of piscivory-associated microbial communities (left) and arthropodophagous-associated microbial communities (right) at the significance level of *p* < 0.01

The amplicon-based inference highlighted the enrichment of multiple pathways involved in the biosynthesis of vitamins (Fig. 6), which was also partly supported by the shotgun approach, and has been also reported in another dietary transition among bats, namely adaptation to sanguivory in vampire bats [14]. Piphillin reported an overall enrichment of pathways involved in the metabolism of vitamins B2, B6 and B9 in piscivorous bats. Enrichment of biosynthesis of vitamins B6 and B9 was not supported by the *M. capaccinii* shotgun data (t-test_B6_: t = 1.32, df = 12.43, *p* value = 0.209; t-test_B9_: t = 0.270, df = 8.79, *p* value = 0.793). However, we found that enrichment of genes involved in the related vitamin B2 biosynthesis was borderline significant (t-test_B2_: t = 2.07, df = 14.65, *p* value = 0.056), and B12 biosynthesis was significantly enriched in *M. capaccinii* (t-test_B12_: t = 3.40, df = 11.75, *p* value = 0.005). The most abundant genes involved in vitamin B12 biosynthesis were assigned to *Aeromonas*, which is the most abundant taxon among *M. capaccinii*. The incidence of *Aeromonas* in *M. pilosus* is much lower, yet they exhibit an increased abundance of *Cetobacterium*, which are also known to produce vitamin B12 [64]. These results suggest that the microbiota of piscivorous bats holds the capacity to synthesize a range of vitamins that contribute to various metabolic and physiological processes.

The analysis of the shotgun sequence data also yielded the enrichment of a number of other metabolic functions observed in the Piphillin-based predictive approach, among which glycan metabolism stood out, with five pathways enriched in piscivorous bats. Glycans are known to play a central role in shaping the composition and activity of intestinal microorganisms, and fish are among the richest sources of certain types of glycans such as glycosphingolipids [65], whose metabolism was also enriched in piscivorous bats. We also detected enrichment of a number of pathways related with lipid metabolism, including alpha-linolenic acid (ALA) and fatty acid degradation that are involved in metabolising omega-3 fatty acids [66]. These types of compounds are highly represented in fish flesh, and they are well-known for their anti-inflammatory activity as rich sources of long-chain fatty acids that are metabolised into short-chain fatty acids (SCFAs) [67].

## Conclusions

Our analysis of the faecal microbiota of the four piscivorous bat species, eleven arthropodophagous counterparts and one fish species consumed by one of the piscivorous bats, enabled us to disentangle the taxonomic and functional microbiota features associated with fishing behaviour in bats. Our results indicate that piscivorous bats carry a microbiota that is distinct to that of arthropodophagous bats. The characteristic bacteria of piscivorous bats likely provide nutritional benefits to their hosts, by synthesizing essential compounds and facilitating the metabolism of complex carbohydrates and lipids acquired from fish and arthropods. The fact that these features are most strongly represented in actively piscivorous bats, yet also present in non-piscivorous colonies of piscivorous bat species, while almost completely absent in arthropodophagous bats, suggests that piscivory-like microbial communities are not a recently acquired trait. In contrast, microbiota fingerprints suggest that the trophic niche expansion produced by incorporating fish into the diet enabled bats to acquire beneficial bacteria otherwise largely inaccessible in the terrestrial environment, which have been established and spread across populations. All in all, our results point to an active contribution of microorganisms to facilitating dietary shifts in vertebrates.

## Methods

### Sampling and sample storage

We captured 98 wild individuals of 15 bat species in 22 localities across Europe, China, Israel, Mexico, and Costa Rica using harp traps and mist nets (see Additional file 1: Table S1.1). The sampled species were *Eptesicus bottae* (Ebo), *Hypsugo ariel* (Har), *Miniopterus schreibersii* (Msc), *Myotis capaccinii* (Mca), *M. daubentonii* (Mda), *M. emarginatus* (Mem), *M. myotis* (Mmy), *M. pilosus* (Mpi), *M. vivesi* (Mvi), *Noctilio leporinus* (Nle), *Pipistrellus kuhlii* (Pku), *Rhinolophus blasii* (Rbl), *R. euryale* (Reu), *R. hipposideros* (Rhi) and *R. ferrumequinum* (Rfe). To avoid sample cross-contamination, each bat was kept separately in a clean, single-use cotton bag for 15–20 min, then identified, sexed and aged before releasing them. Faecal pellets were collected from the bags and stored in 1.5 ml collection tubes filled with ethanol.

For the analysis of fish microbiota, 13 individuals of the exotic fish *Gambusia holbrooki* were captured using minnow trap nets at four wetlands in south-eastern Spain, where the piscivorous colony of *Myotis capaccinii* is located. Fish were kept in containers with water from the sample location, which were continually oxygenated to prevent hypoxia. Within a maximum period of four hours after sampling, fish were euthanized by a quick blow to the head in compliance with the Spanish law on animal research ethics (RD 53/2013) and the European Directive on the protection of animals used for scientific purposes (2010/63/EU). The entire gastrointestinal tract was removed from the fish using scalpels, after which the gut content was separated from the intestine and stored in 1.5 ml collection tubes filled with ethanol.

All samples were refrigerated (4-8ºC) until they were transported to the laboratory, after which they were stored at -20ºC until DNA extraction. All captures were authorised by the competent authorities of the countries in which they were carried out.

### Morphological analysis of bulk guano samples

Bulk faecal material was collected from the roosting caves of *M. capaccinii*. In the laboratory each bulk was homogenized with water and then filtered through two laboratory sieves with different mesh sizes (2 mm and 0.5 mm). The unfiltered material was inspected by magnifying lens for fish remains, such as otoliths, bones and scales.

### Laboratory work

#### DNA extraction

DNA was extracted in a dedicated pre-PCR laboratory following a randomised setup from 1–3 bat droppings (ca. 20 mg) of each bat individual and the entire gut content of the fish. The PowerSoil® DNA Isolation Kit (MoBio, CA, USA) was used following the manufacturer’s protocol (2016 version) with the modifications explained in Alberdi et al. [68]. Each extraction round included 23 samples and one negative extraction control. The final DNA extracts (50 µl) were aliquoted in five subsamples to avoid any potential contamination of the entire volume during later processing.

#### 16S amplicon sequencing

The V3 and V4 hypervariable regions of the bacterial 16S rRNA were targeted using the 341F/806R primer pair [69, 70]. Prior to the tagged PCRs, the most optimal annealing temperature was assessed by amplifying five extracts and one extraction blank using temperatures ranging from 50ºC to 62ºC. Additionally, quantitative PCR (qPCR) screening was carried out with multiple DNA template volumes on six extracts and both extraction blanks to (1) assess contamination of extraction blanks, (2) determine the optimal cycle number for the subsequent PCRs, and (3) estimate the maximum template amount for the following tagged PCR amplifications in which PCR inhibitory substances, copurified with the DNA, would not distort the amplification [71–73]. PCRs were run on an Applied Biosystems 2720 Thermal Cycler with a reaction volume of 25 μl. Each PCR reaction contained 2.5 μl of AmpliTaq Gold buffer (final concentration 1X), 2.5 μl of MgCl_2_ (2.5 mM), 1.5 μl of BSA (1.2 ng/μl), 0.5 μl of dNTP (0.2 mM), 0.5 μl of AmpliTaq Gold DNA polymerase (0.1 U/μl), 2 μl of primer mix (0.8 mM) and 13.5 μl of ddH_2_O. PCR settings were 95°C for 10 min, 30 cycles of 95°C for 15 s, 53°C for 20 s and 72°C for 40 s and at last 72°C for 10 min. The PCR products from different samples were pooled in batches of 24 samples while making sure that each sample had its own unique tagged primer set within the pool, in order to enable tracking the sample back to the individual of origin [74]. All amplicon pools were subsequently purified at 1:1 beads:DNA volume-ratio to remove non-target DNA and primer dimers using SPRI beads [75, 76]. Sequencing libraries were prepared using the Tagsteady library building protocol [77], and sequenced on an Illumina MiSeq machine using 250PE chemistry.

#### Shotgun sequencing

A subset of DNA extracts was also processed for shotgun sequencing. Specifically, 18 samples belonging to 7 arthropodophagous and 11 piscivorous bat individuals were sonicated into fragment-lengths around 350 bp on a Covaris S220x Focused ultrasonicator (Covaris Inc., Woburn, MA). Fragmented DNA (200 ng) was built into shotgun sequencing libraries using the BEST protocol, as described in [78]. Libraries were purified using SPRI beads (1:1.4 DNA:beads ratio) followed by a qPCR screening to determine the optimal number of cycles for PCR indexing with different reverse indices. Indexing cycle numbers adjusted to the molarity of each library were in the range of 7–16. After indexing, an additional purification step was performed using SPRI beads (1:1.2 DNA:beads ratio) before pooling multiple libraries for multiplex sequencing in an Illumina HiSeq2500 platform with 150PE chemistry.

### Data analysis

#### Bioinformatic processing of amplicon data

Amplicon sequencing reads were demultiplexed based on library indices using AdapterRemoval [79]. As the library building approach we used is based on adapter ligation rather than PCR amplification, the resulting DNA sequences can be either in Forward-Reverse or Reverse-Forward direction. Using Cutadapt 1.18 [80], we identified primer locations, and reverse complemented the reads in Reverse-Forward direction to ensure unidirectionality of all sequences. Taxonomic assignment was done by the naive Bayesian classifier method with default settings as implemented in DADA2 [81] in R 3.6.1 [82], against SILVA 16S rRNA gene reference taxonomy database. The initial ASV table was generated for the 15,856 ASVs that were taxonomically annotated.

Contamination filtering was independently performed in each batch of samples with its corresponding controls using decontam (Davis et al., 2018) [99]. The 18 putative contaminants were removed from the ASV table. Rarefaction curves were plotted using the R package vegan to identify samples not reaching a diversity saturation plateau in the number of sequencing reads. Based on this analysis, six samples with fewer than 10,000 reads were removed from the dataset. To minimise impact of potential false positives, ASVs with less copies than 0.01% of the total number of reads of each sample were removed, which reduced the ASV table to 9298 ASVs. Finally, the ASVs that could not be taxonomically identified as Bacteria (129 ASVs) or to Phylum and Class level (37 ASVs) were removed and the output ASV table (9132 ASVs) was used for downstream analyses.

#### Functional prediction from amplicon data

Functional prediction of the microbial content from 16S rRNA gene sequencing data was performed using Piphillin [63]. Piphillin predicts metagenomic content via direct nearest-neighbor matching between 16S rRNA gene amplicons and genomes from KEGG reference database. The analysis was run using a 99% ID cutoff [83]. Only metabolic pathways were included in the differential abundance analysis. The differential abundance analysis in the functionality of the gut microbiota of arthropodophagous and piscivorous bats was performed using the univariate DESeq2 method [84]. DESeq2 was based on the negative binomial Wald test and parametric fitType. Only ASVs with False Discovery Rate adjusted *p* value < 0.01 were considered statistically significant. ggplot() function of the R package *tidyverse* was used for data visualization [85].

#### Ensemble machine learning-based modelling

We implemented an ensemble approach consisting of three machine learning algorithms, namely logistic regression, Random Forest and XGBoost, to first gain further insights into the compositional differences of the gut microbiota of arthropodophagous and piscivorous bats, and second, to ascertain the classification into arthropodophagous-like or piscivorous-like microbiotas in the three *M. capaccinii* populations with “uncertain” dietary preferences. We used a set of 84 samples (47 arthropodophagus and 37 piscivorous) for training the models using the caretList function in the R library caretEnsemble [19, 86]. ROC was used to select the optimal model using the largest value, and arthropodophagus was used as the ‘positive’ class to compute sensitivity (Sens) and specificity (Spec) values [87]. We used caretStack to merge the models into a predictive meta-model. Feature (ASV) importance for the classification using the meta-model was obtained using the function varImp. The ensemble model was used to predict the classification of 15* M. capacinii* from allegedly non-piscivorous colonies using the function predict, after which prediction statistics were obtained using the confusionMatrix and custom functions.

#### Diversity and compositional analysis

All the analyses were carried out in the R statistical environment [82] and the diversity analyses were performed based on the relative abundance of each ASV (calculated as ASV read depth over total read depth per library). Diversity analyses and visualization were carried out using the div_test and div_test_plot function of the R package *hilldiv* based on abundance-based Hill numbers [18]. The maximum likelihood phylogeny generated from ASV sequences using RAxML-NG was employed for phylogenetic diversity metrics. Richness was computed as the neutral Hill number of order of diversity q = 0; richness and evenness was computed as the neutral Hill number of order of diversity q = 1―i.e. Shannon diversity― and richness, evenness and regularity was computed as the phylogenetic Hill number of order of diversity q = 1 [88]. Diversity comparisons between species were carried out using the Kruskal–Wallis (K-W) rank sum test, followed by a *posthoc* Dunn's test with Bonferroni-corrected *p* values. The Jaccard-type turnover-complement (SqN) was computed using beta_dis() function of package *hilldiv*. The function was run by inputting values from the object outputted by the div_part() function.

Compositional differences were computed by pairwise distances among samples using the pair_dis function of the R package *hilldiv* based on abundance-based Hill numbers [18]. Compositional differences were contrasted using permutational multivariate analyses of variance (PERMANOVA) with 999 permutations using the vegan::adonis function [89]. calc_pairwise_permanovas function of R package *mctoolsr* was used to calculate pairwise post-hoc comparisons between samples [90]. The level of homogeneity of dispersion within groups was first analysed using vegan::betadisper and vegan::permutest function (*p* value > 0.05, see Additional file 1: Table S1.4) [89]. Since this assumption was not met when richness was computed, in the results section only NMDS and PERMANOVA based on pairwise distances calculated through the neutral Hill number of order of diversity q = 1 (PERMANOVA_U12_) and phylogenetic Hill number of order of diversity q = 1 (PERMANOVA_Ū12_) are shown. Statistically significant results were considered at *p* values < 0.05, which were adjusted for False discovery rate (*p* value_FDR_) (Benjamini and Hochberg 1995) [100]. The dis_nmds function of package *hilldiv* was used for plotting the NMDSs. The core microbiota was explored using the *microbiome* package [91]. The detection prevalence of 90% across samples was setted up. We analysed the core microbiota of all studied bats together as well as arthropodophagous and piscivorous bat species separately. A differential abundance analysis using the univariate DESeq2 method [84] was performed to identify the individual ASVs driving the changes between arthropodophagous and piscivorous bats.

#### Bioinformatic processing of shotgun data

Shotgun sequencing reads were demultiplexed and quality-filtered using AdapterRemoval. Duplicated reads were filtered using seqkit 0.7.1 [92] and bat and fish DNA was removed from the dataset using bwa mem [93] by mapping the reads against the reference genome sequences of the closest relative of *Myotis capaccinii*, namely *M. myotis* and the draft genome of *Gambusia holbrooki*, respectively. Presence of fish remains in metagenomic data was assessed through analysing the mapping rate to the *G. holbrooki* genome. Due to conserved genomic regions across vertebrates, mapping rate to fish genomes even in the absence of fish remains is not zero, but oscillates at a basal level of a few sequences per million reads. Hence, the threshold we employed to consider fish DNA remains were actually detected was three standard deviations larger than the average mapping rate of arthropodophagous bats to the *G. holbrooki* genome. Subsequently, we mapped the preprocessed reads against the reference genomes of *Aeromonas veronii* (ASM869370v1) and used the software iRep to estimate bacterial replication rates by means of the peak-to-trough ratio (PTR) [94, 95]. Metagenomic reads were then co-assembled using Megahit [96] and open reading frames (ORF) predicted using Prodigal 2.6.3 [97]. ORFs were functionally annotated by aligning them against the KEGG database using GhostKoala [98], and the reads of each sample mapped to the ORF catalogue to obtain the overall functional profile of the microbial metagenome. To analyse the presence of enterotoxin-encoding genes, we aligned the ORF sequences to a custom reference database of 86 amino acid sequences of *Aeromonas* enterotoxic genes created using sequences available at Uniprot. Then, reads of each sample were mapped to the *Aeromonas* enterotoxic genes to obtain the depth of coverage of such genes. Statistical comparisons were made with Student t-test in R.

## Supplementary Information

**Additional file 1**. Supplementary figures and tables.

## Data Availability

The raw data have been deposited at the NCBI SRA database under the project accession number PRJEB47836. Bioinformatics pipelines used to process the sequence data and generate count tables are available at https://github.com/ostaizka/fishing-bats.
