# Supplementary material for: The role of the gut microbiota in the dietary niche expansion of fishing bats
Source: Anim Microbiome. 2021 Oct 28;3:76. doi: 10.1186/s42523-021-00137-w (PMC8555116; doi:10.1186/s42523-021-00137-w)
Supplement: Supplementary file 1 — Additional file 1. Supplementary figures and tables. [file 42523_2021_137_MOESM1_ESM.docx]

The role of the gut microbiota in the dietary niche expansion of fishing bats

Ostaizka Aizpurua^1^, Lasse Nyholm^1^, Evie Morris^2^, Gloriana Chaverri^3,4^, Luis Gerardo Herrera Montalvo^5^, José Juan Flores-Martinez^6^, Aiqing Lin^7^, Orly Razgour^2^, M Thomas P Gilbert^1,8^, Antton Alberdi^1^

**Table S1.1.** Arthropodophagous and piscivorous bat species included in the study.

| **Species** | **Family** | **Localities** | **Habitat** | **Diet** |
| --- | --- | --- | --- | --- |
| *Eptesicus bottae* | Vespertilionidae | 1 | Freshwater | Arthropods |
| *Hypsugo ariel* | Vespertilionidae | 1 | Freshwater | Arthropods |
| *Miniopterus schreibersii* | Vespertilionidae | 2 | Freshwater | Arthropods |
| *Myotis capaccinii* | Vespertilionidae | 4 | Freshwater | Arthropods & Fish |
| *Myotis daubentonii* | Vespertilionidae | 1 | Freshwater | Arthropods |
| *Myotis emarginatus* | Vespertilionidae | 1 | Freshwater | Arthropods |
| *Myotis myotis* | Vespertilionidae | 2 | Freshwater | Arthropods |
| *Myotis pilosus* | Vespertilionidae | 1 | Freshwater | Arthropods & Fish |
| *Myotis vivesi* | Vespertilionidae | 1 | Ocean | Arthropods & Fish |
| *Noctilio leporinus* | Noctilionidae | 1 | Brackish | Arthropods & Fish |
| *Pipistrellus kuhlii* | Vespertilionidae | 1 | Freshwater | Arthropods |
| *Rhinolophus blasii* | Rhinolophidae | 1 | Freshwater | Arthropods |
| *Rhinolophus euryale* | Rhinolophidae | 1 | Freshwater | Arthropods |
| *Rhinolophus ferrumequinum* | Rhinolophidae | 1 | Freshwater | Arthropods |
| *Rhinolophus hipposideros* | Rhinolophidae | 1 | Freshwater | Arthropods |

**Figure S1.1:** Barplot of relative abundances of bacterial families found in the fecal communities of all studied bats.


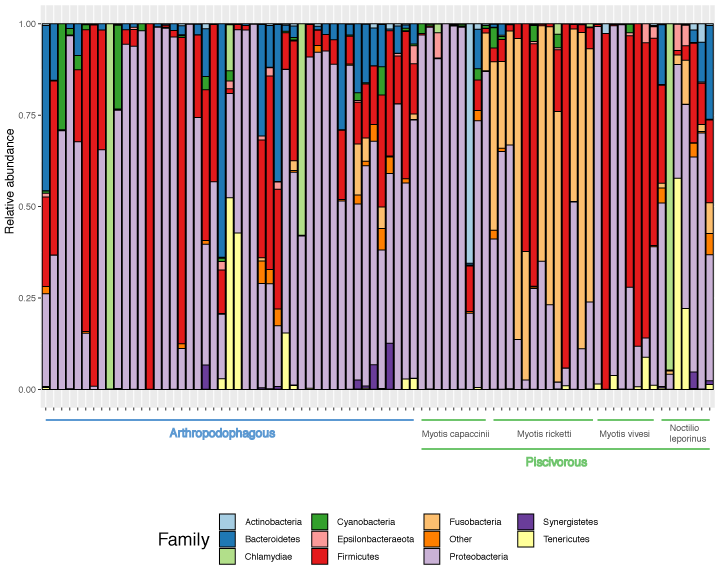


**Figure S1.2.** Alpha diversity values of ASV-level bacterial communities from piscivorous and arthropodophagous bats computed as the neutral and phylogenetic Hill numbers of order of diversity q = 0 (A-B) and q = 1 (C-D); each point represents a sample. Wilcoxon Rank Sum Test test was performed to compare alpha diversity estimates between the two groups. None of the comparisons was statistically significant (p < 0.05, the specific values can be found in Table S1.2).

A B


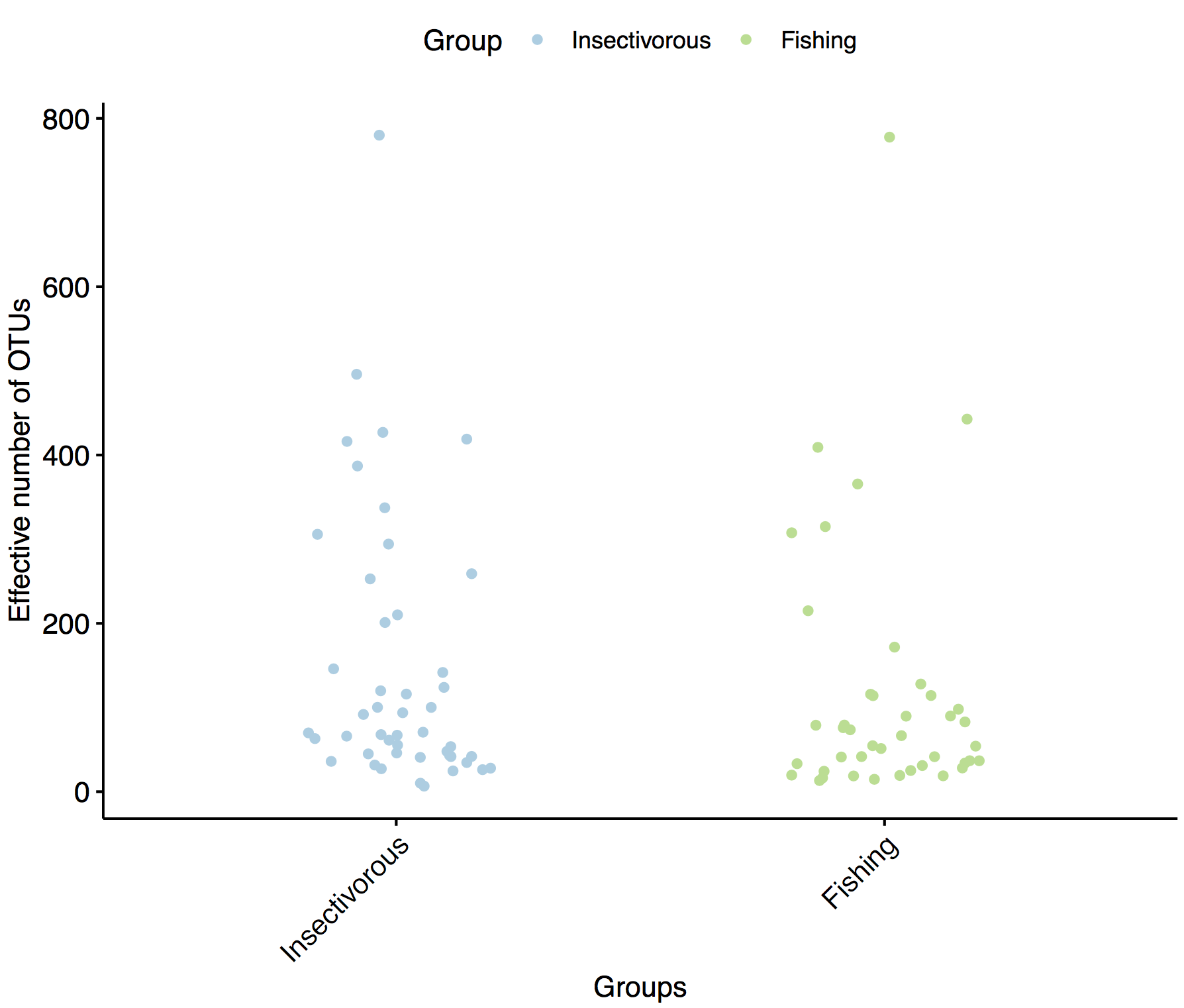

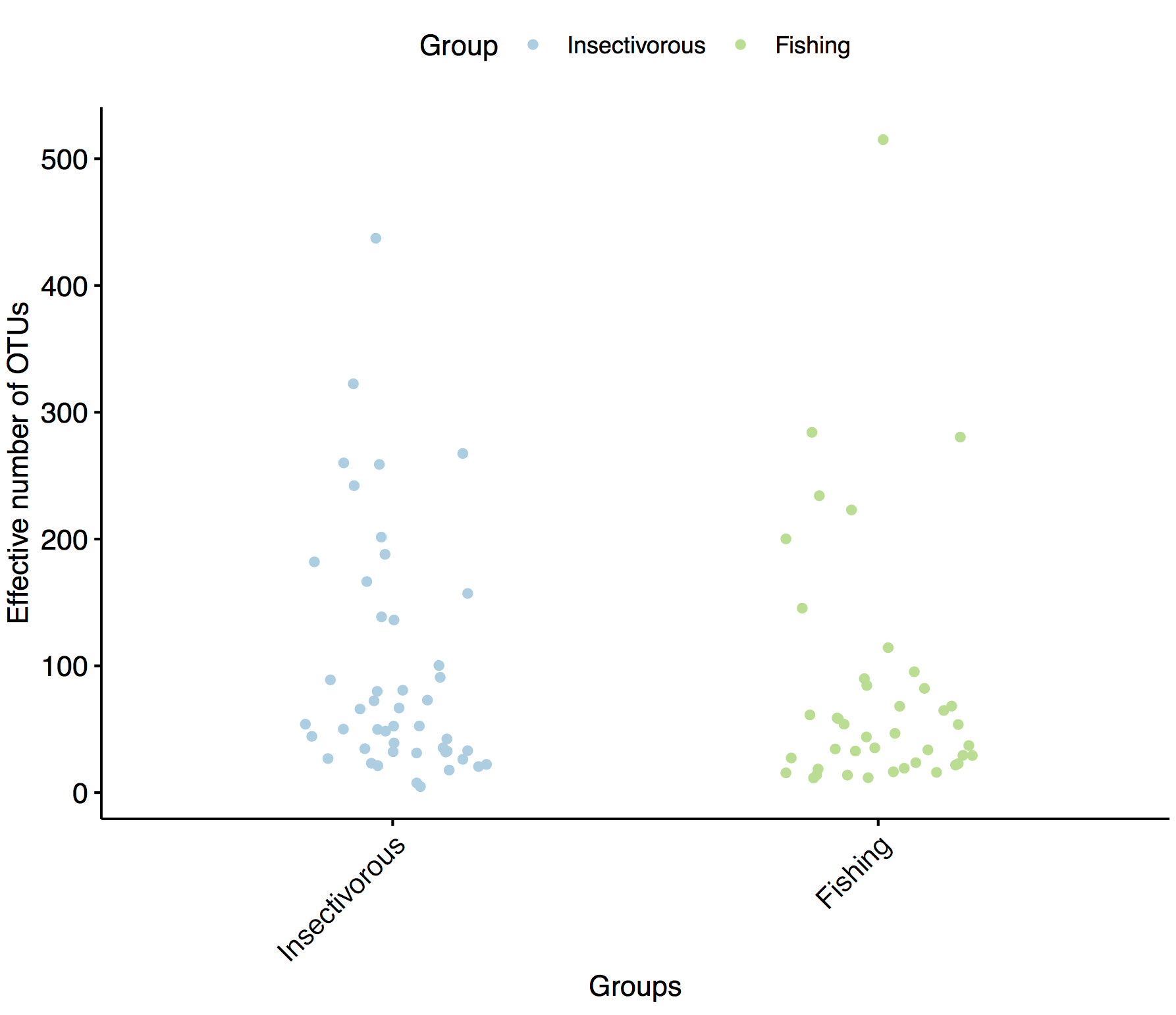


C D


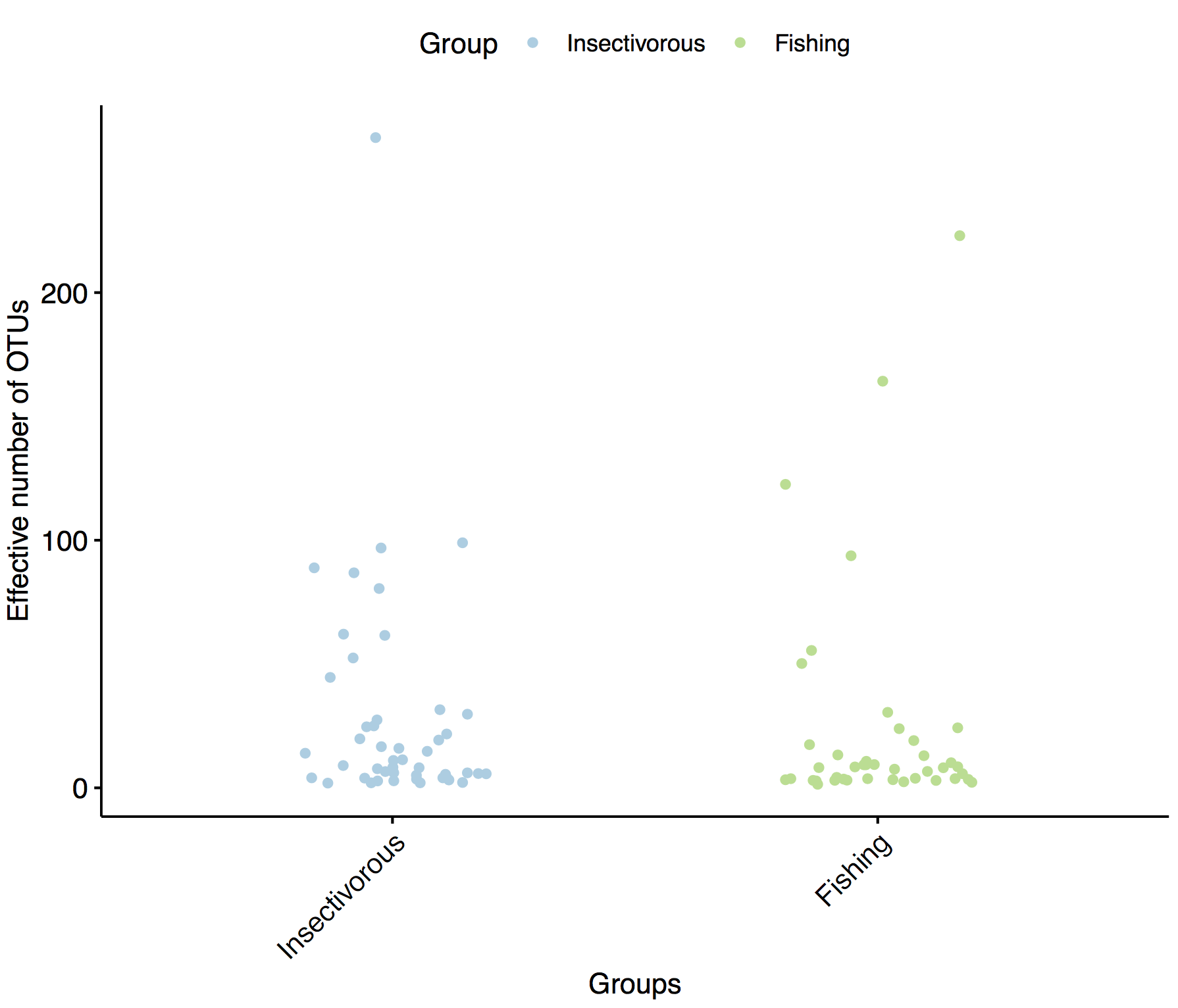

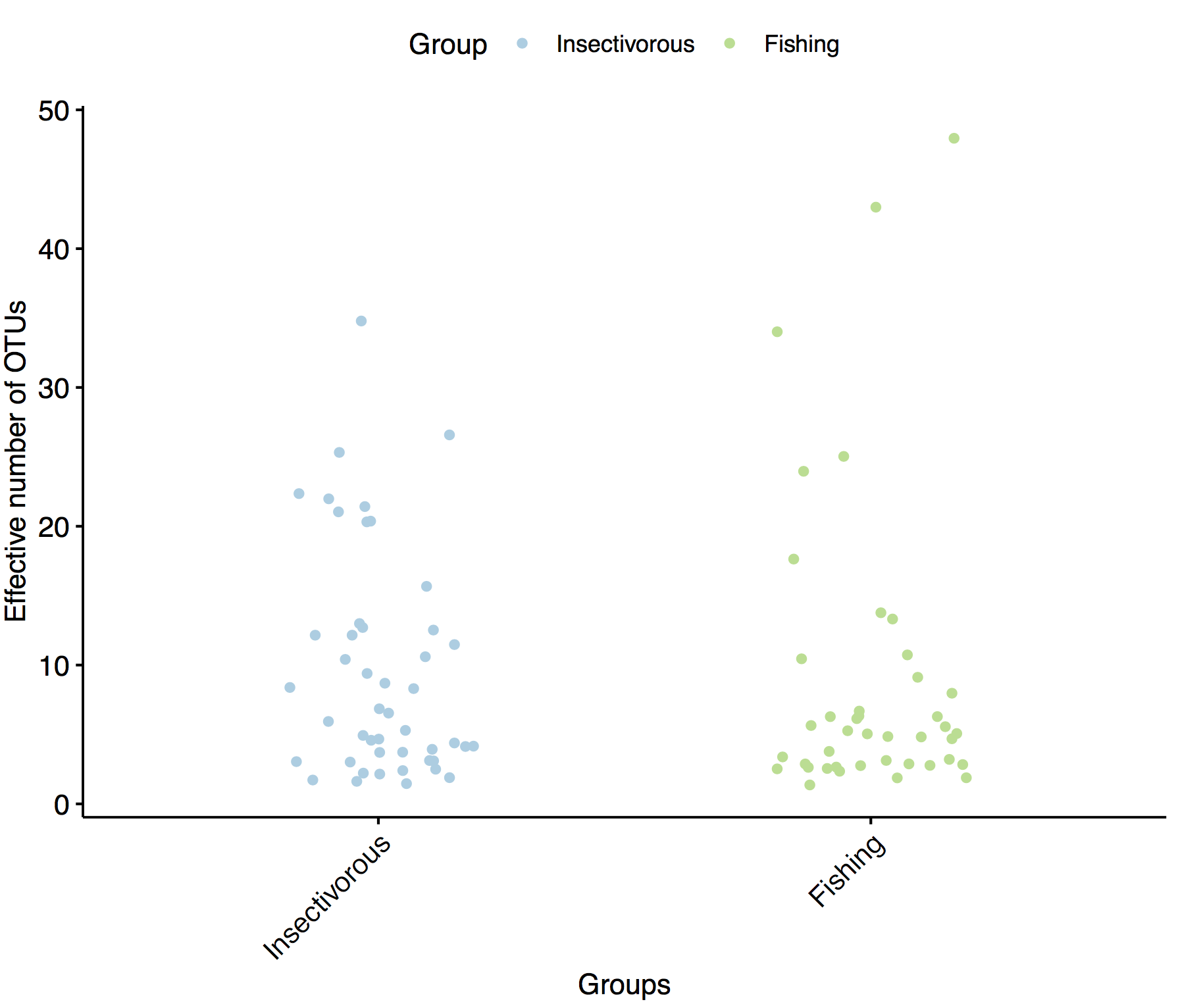


**Table S1.2.** Alpha diversity metrics computed as the neutral and phylogenetic Hill number of order of diversity q = 0 and q = 1 values of ASV-level bacterial communities from piscivorous and arthropodophagous bats. A) Values obtained from Wilcoxon Rank Sum Test and B) gut microbial diversity values (mean ± standard deviation).

A)

| **q value** | **Type of Hill number** | **Normality p-value** | **Homogeneity p-value** | **W** | **P-value** |
| --- | --- | --- | --- | --- | --- |
| 0 | Neutral | 3.393e-11 | 0.0024 | 667.5 | 0.0844 |
| 1 | Neutral | 8.7447e-15 | 0.7261 | 668 | 0.0699 |
| 0 | Phylogenetic | 2.6477e-11 | 0.7660 | 832 | 0.2052 |
| 1 | Phylogenetic | 5.7172e-11 | 0.0637 | 871 | 0.3442 |

B)

|  | **Neutral Hill number, q = 0** | **Neutral Hill number, q = 1** | **Phylogenetic Hill number, q = 0** | **Phylogenetic Hill number, q = 1** |
| --- | --- | --- | --- | --- |
| Arthropodophagous | 147.38±161.68 | 28.28±44.67 | 96.02±95.22 | 9.59±8.10 |
| Piscivorous | 64.30±61.73 | 20.15±42.25 | 80.76±99.64 | 8.93±10.76 |

**Table S1.3**. The top ten ASVs that contributed the most to the ensemble predictive models.

| **ASV** | **overall** | **rf** | **lr** | **xgb** | **Taxon names** |
| --- | --- | --- | --- | --- | --- |
| ASV_1 | 4.170 | 7.707 | 0.778 | 20.792 | *Aeromonas* |
| ASV_40 | 3.952 | 6.609 | 0.892 | 21.727 | *Aeromonas* |
| ASV_23 | 3.357 | 5.020 | 0.425 | 24.985 | *Photobacterium* |
| ASV_2 | 1.611 | 3.654 | 0.637 | 1.043 | *Cetobacterium* |
| ASV_158 | 1.505 | 1.916 | 0.297 | 11.831 | *Unknown Pasteurellaceae* |
| ASV_167 | 1.272 | 2.545 | 0.297 | 4.723 | *Mycoplasma* |
| ASV_30 | 1.147 | 2.479 | 0.552 | 0.368 | *Paraclostridium* |
| ASV_58 | 1.039 | 2.208 | 0.552 | 0.00 | *Cetobacterium* |
| ASV_282 | 0.993 | 2.127 | 0.297 | 2.292 | *Cardiobacterium* |
| ASV_54 | 0.906 | 1.827 | 0.510 | 0.221 | *Plesiomonas* |

**Figure S1.3.** Dissimilarity metrics computed as the neutral and phylogenetic Hill number of order of diversity q = 0 and q = 1 values are represented as a network. Each species is a node and each distance an edge; in which the width of the edges represent the magnitude of the correlation.


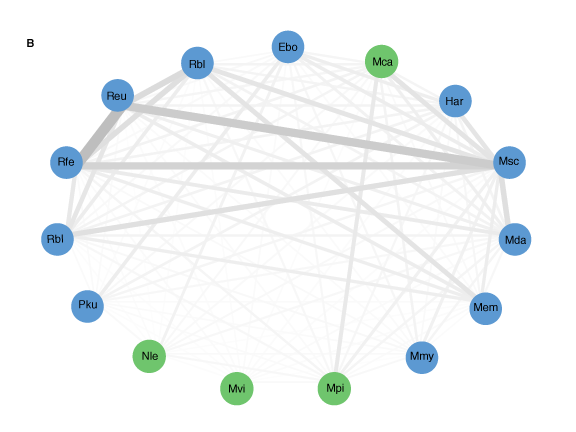

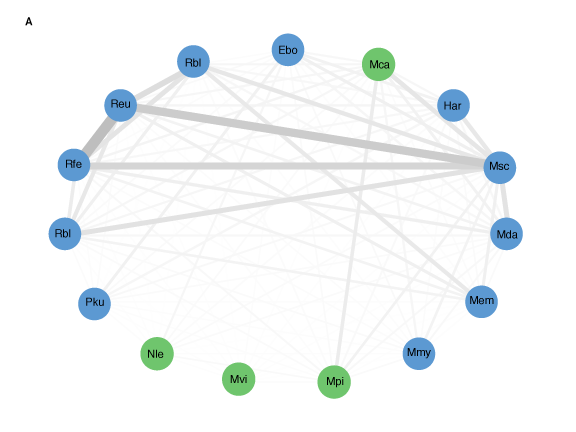


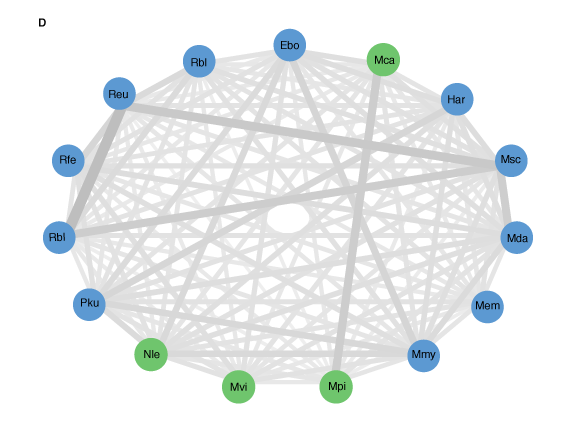

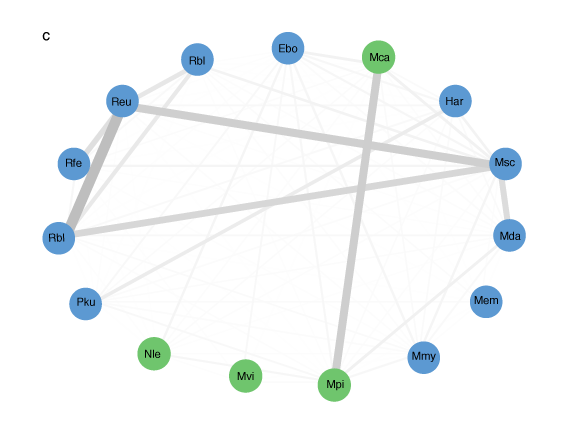


**Table S1.4:** Results from the A) Permutest and B) PERMANOVA analysis using dissimilarity distances computed as the neutral and phylogenetic Hill number of order of diversity q = 0 and q = 1 values.

A)

|  | **Neutral Hill number, q = 0** | | **Neutral Hill number, q = 1** | | **Phylogenetic Hill number, q = 0** | | **Phylogenetic Hill number, q = 1** | |
| --- | --- | --- | --- | --- | --- | --- | --- | --- |
|  | F | P-value | F | P-value | F | P-value | F | P-value |
| Diet | 2.7291 | 0.095 | 5.700 | 0.027 | 2.6261 | 0.101 | 1.1473 | 0.282 |
| Habitat | 32.306 | 0.001 | 7.088 | 0.003 | 29.945 | 0.001 | 15.116 | 0.001 |
| Family | 24.613 | 0.001 | 4.598 | 0.015 | 29.155 | 0.001 | 7.5552 | 0.004 |
| Species | 5.435 | 0.001 | 0.760 | 0.715 | 4.0183 | 0.001 | 0.7999 | 0.663 |

B)

|  | **Neutral Hill number, q = 0** | | **Neutral Hill number, q = 1** | | **Phylogenetic Hill number, q = 0** | | **Phylogenetic Hill number, q = 1** | |
| --- | --- | --- | --- | --- | --- | --- | --- | --- |
|  | R^2^ | P-value | R^2^ | P-value | R^2^ | P-value | R^2^ | P-value |
| Diet | 0.0298 | 0.001 | 0.0567 | 0.001 | 0.0320 | 0.001 | 0.0720 | 0.001 |
| Habitat | 0.0218 | 0.001 | 0.0496 | 0.001 | 0.0298 | 0.003 | 0.0403 | 0.003 |
| Family | 0.0347 | 0.001 | 0.0496 | 0.001 | 0.0167 | 0.001 | 0.0252 | 0.001 |
| Species | 0.1736 | 0.001 | 0.1954 | 0.001 | 0.1713 | 0.001 | 0.1978 | 0.001 |

**Figure S1.4:** NMDS computed as the neutral and phylogenetic Hill number of order of diversity q = 0 (A-B) and q = 1 (C-D) values of ASV-level bacterial communities of piscivorous bats (*Myotis capaccinii*, *M. pilosus*, *M. vivesi* and *Noctilio leporinus*).

A B


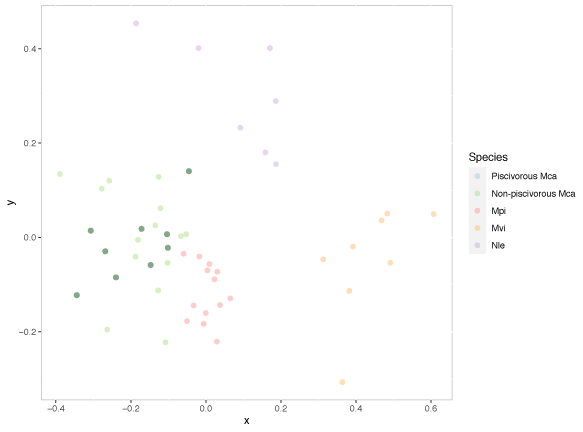

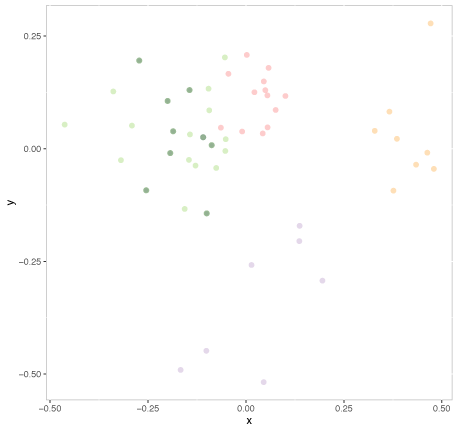


C D


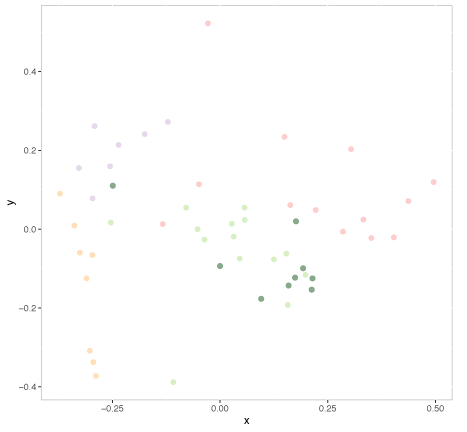


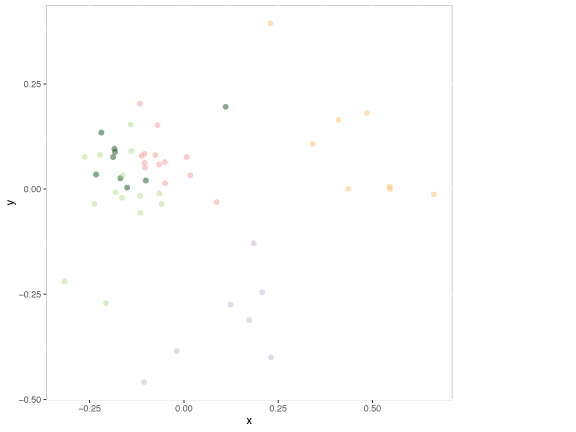


**Table S1.5.** Results from the A) Permutest and B) PERMANOVA analysis using dissimilarity distances computed as the neutral and phylogenetic Hill number of order of diversity q = 0 and q = 1 values in piscivorous bats (*Myotis capaccinii, M. pilosus, M. vivesi, N. leporinus*). C) Pairwise comparison at neutral Hill number q = 1.

| **Permutest** | **Neutral Hill number, q = 0** | | **Neutral Hill number, q = 1** | | **Phylogenetic Hill number, q = 0** | | **Phylogenetic Hill number, q = 1** | |
| --- | --- | --- | --- | --- | --- | --- | --- | --- |
|  | F | P-value | F | P-value | F | P | P-value | P-value |
| Species | 4.197 | 0.007 | 1.483 | 0.236 | 4.278 | 0.008 | 0.729 | 0.562 |

| **Adonis** | **Neutral Hill number, q = 0** | | **Neutral Hill number, q = 1** | | **Phylogenetic Hill number, q = 0** | | **Phylogenetic Hill number, q = 1** | |
| --- | --- | --- | --- | --- | --- | --- | --- | --- |
|  | R^2^ | P-value | R^2^ | P-value | R^2^ | P-value | R^2^ | P-value |
| Species | 0.1667 | 0.001 | 0.312 | 0.001 | 0.174 | 0.001 | 0.345 | 0.001 |

| **Neutral Hill number, q = 1** | | | | |
| --- | --- | --- | --- | --- |
| X1 | X2 | R^2^ | P-value | P-value_FDR_ |
| Piscivorous *M. capaccinii* | Non-piscivorous *M. capaccinii* | 0.065 | 0.090 | 0.090 |
| Piscivorous *M. capaccinii* | *M. pilosus* | 0.210 | 0.001 | 0.002 |
| Piscivorous *M. capaccinii* | *M. vivesi* | 0.328 | 0.002 | 0.002 |
| Piscivorous *M. capaccinii* | *N. leporinus* | 0.269 | 0.001 | 0.002 |
| Non-piscivorous *M. capaccinii* | *M. pilosus* | 0.174 | 0.001 | 0.002 |
| Non-piscivorous *M. capaccinii* | *M. vivesi* | 0.233 | 0.001 | 0.002 |
| Non-piscivorous *M. capaccinii* | *N. leporinus* | 0.176 | 0.001 | 0.002 |
| *M. pilosus* | *M. vivesi* | 0.323 | 0.001 | 0.002 |
| *M. pilosus* | *N. leporinus* | 0.256 | 0.001 | 0.002 |
| *M. vivesi* | *N. leporinus* | 0.210 | 0.002 | 0.002 |

**Figure S1.5.** Differentially abundant ASVs between *Myotis capaccinii* colony and two arthropodophagous bats species that share the roost with the former displayed at the genus level. Only ASVs with a significance of *p* < 0.01 are shown. A lower than zero log2-fold-change indicates phyla that were more differentially abundant in *M. capaccinii* bats. Each circle represents a single ASV, thus multiple circles within a genus indicate multiple ASV that were enriched.


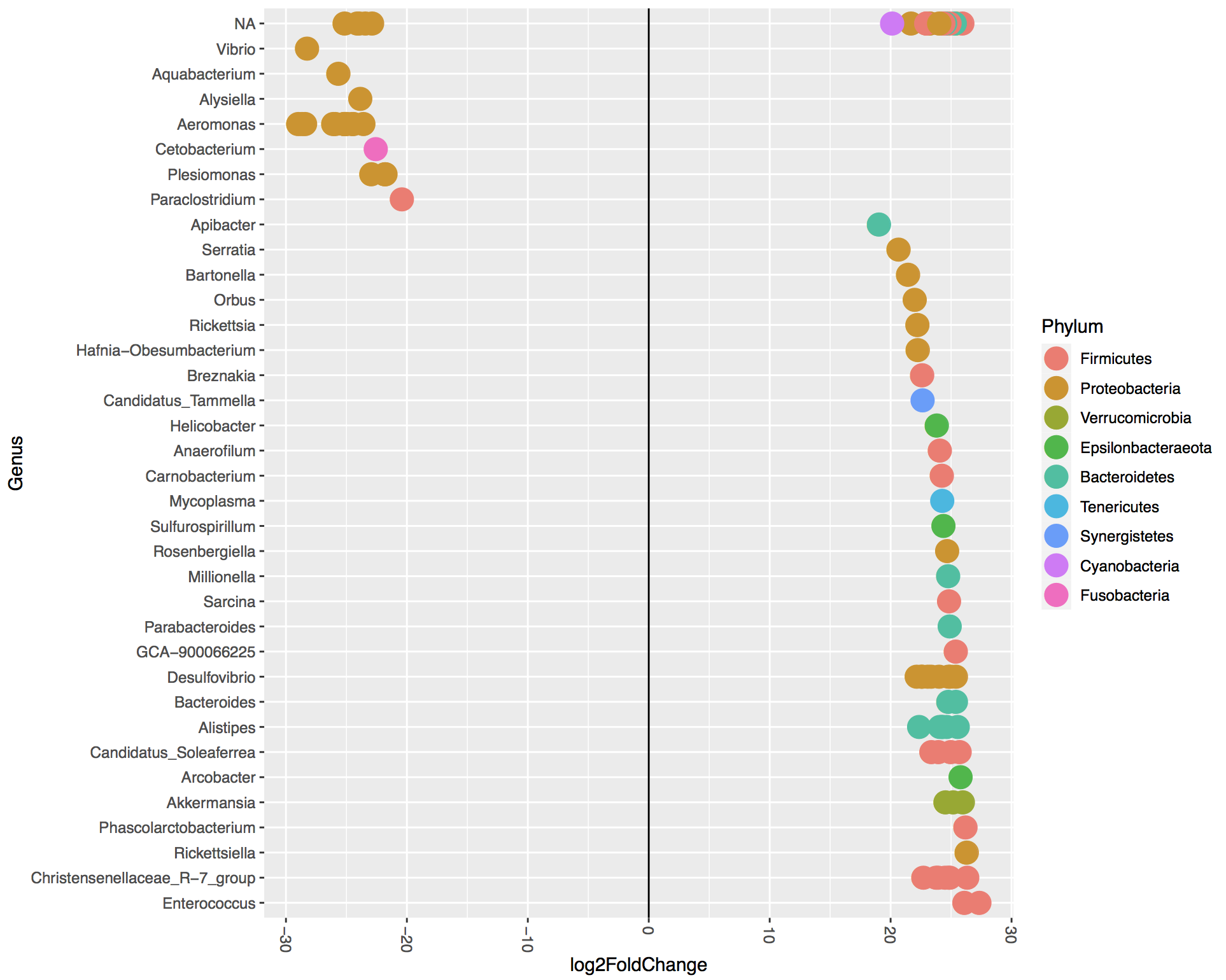


**Table S1.6.** Fish detection and bacteria replication rate analyses based on shotgun metagenomic data. Species other than *Myotis capaccinii* were used as reference for measuring the background mapping noise and set the fish detection threshold (see Methods for details). ORI and TER values indicate the normalised coverage values at the replication origin and terminus regions, while PTR shows the peak-to-trough ratio that estimates bacterial replication rate.

| **ID** | **Species** | **Colony** | **Fish per million reads** | **Fish detected** | **ORI** | **TER** | **PTR** | **Note** |
| --- | --- | --- | --- | --- | --- | --- | --- | --- |
| ES166 | *Rhinolophus ferrumequinum* | Other | 496 | Reference | NA | NA | NA | Too little coverage for PTR analysis |
| ES172 | *Myotis emarginatus* | Other | 134 | Reference | NA | NA | NA | Too little coverage for PTR analysis |
| ES174 | *Rhinolopus euryale* | Other | 548 | Reference | NA | NA | NA | Too little coverage for PTR analysis |
| ES190 | *Miniopterus schreibersii* | Other | 414 | Reference | NA | NA | NA | Too little coverage for PTR analysis |
| ES244 | *Myotis daubentonii* | Other | 962 | Reference | NA | NA | NA | Too little coverage for PTR analysis |
| ES264 | *Myotis daubentonii* | Other | 859 | Reference | NA | NA | NA | Too little coverage for PTR analysis |
| ES354 | *Miniopterus schreibersii* | Other | 1784 | Reference | NA | NA | NA | Too little coverage for PTR analysis |
| ES365 | *Myotis capaccinii* | Non-fishing | 261 | No | NA | NA | NA | Too little coverage for PTR analysis |
| ES437 | *Myotis capaccinii* | Non-fishing | 492 | No | 3241229 | 873884 | 3.29 |  |
| ES441 | *Myotis capaccinii* | Non-fishing | 531 | No | 3225220 | 848672 | 2.8 |  |
| ES466 | *Myotis capaccinii* | Fishing | 95383 | Yes | 3292080 | 783051 | 1.87 |  |
| ES468 | *Myotis capaccinii* | Fishing | 118213 | Yes | 3.231.695 | 845959 | 2.42 |  |
| ES470 | *Myotis capaccinii* | Fishing | 748 | No | 3.262.612 | 847967 | 3.19 |  |
| ES472 | *Myotis capaccinii* | Fishing | 1017 | No | 3.237.800 | 857097 | 3.18 |  |
| ES610 | *Myotis capaccinii* | Non-fishing | 780 | No | 3239755 | 863977 | 3.33 |  |
| ES612 | *Myotis capaccinii* | Non-fishing | 540 | No | 3228061 | 857058 | 2.96 |  |
| ES619 | *Myotis capaccinii* | Fishing | 72885 | Yes | 3217332 | 859580 | 2.92 |  |
| ES621 | *Myotis capaccinii* | Fishing | 1868 | No | NA | NA | NA | Too little coverage for PTR analysis |
